# Bibliography

**Published:** 1900-08

**Authors:** 


### Bibliography.

Annual and Analytical Cyclopædia of Practical Medicine. By Charles E. de M. Sajous, M.D., and one hundred Associate Editors, assisted by Corresponding Editors, Collaborators, and Correspondents. Illustrated with Chromo-Lithographs, Engravings, and Maps. Volume V. The F. A. Davis Company, Publishers, Philadelphia, New York, Chicago, 1900.

The fifth volume of this Cyclopædia fully maintains the high character of those that have preceded it. The rapidity with which these volumes have been produced and the care manifested upon every page indicate an energetic and intelligent labor that all must appreciate. The editor says of it, "It has proved to be the most arduous one to prepare of the entire series, involving, as it does, almost every specialty,—otology, laryngology, ophthalmology, neurology, pædiatrics, obstetrics, therapeutics, etc., besides the sections usually classed under general medicine and surgery."

The present volume opens under the letter M with "Methyl-Blue" and closes with "Rabies," and covers between these two subjects, through six hundred and sixty-two pages, a series of valuable and thorough papers. One of the most valuable, especially to dentists, is the article on "Nursing and Artificial Feeding." The importance of this not only to the health of the infant, but as preparatory to that troublesome period known as dentition, can hardly be over-estimated. While this does not enter into the treatment of the subject-matter, the whole question of proper feeding is so intimately connected with the evolution of the dental organs that the dental practitioner cannot fail to appreciate this admirable treatise. It is confined to the first year of the infant's life, but this is the important period, and if the instructions given were followed this dangerous period would be passed with less difficulty than is usually experienced where little or no attention is paid to rules of feeding.

The various notices of these volumes, given in this journal as they have appeared, have presented but one opinion as to their value, and these favorable expressions can be repeated here. They have all aimed to give an exhaustive treatment of the various subjects, and have left little to be desired as works of reference. To the busy general practitioner or specialist they have presented full, compact and reliable information, readily obtainable without wearying search through a multitude of authorities. The practice of medicine has become so extended, branching more and more into specialties, that it has become a practical impossibility for even the most learned to keep in step with its progress in all its relations. This work, therefore, simply fills a great need, and becomes an essential part of every professional library.

This volume has been prepared by the publishers with the same careful attention to details as those preceding, leaving nothing to be desired in this respect.

